# Activation of the Cellular Unfolded Protein Response by Recombinant Adeno-Associated Virus Vectors

**DOI:** 10.1371/journal.pone.0053845

**Published:** 2013-01-08

**Authors:** Balaji Balakrishnan, Dwaipayan Sen, Sangeetha Hareendran, Vaani Roshini, Sachin David, Alok Srivastava, Giridhara R. Jayandharan

**Affiliations:** 1 Department of Hematology, Christian Medical College, Vellore, Tamil Nadu, India; 2 Centre for Stem Cell Research, Christian Medical College, Vellore, Tamil Nadu, India; University of Florida, United States of America

## Abstract

The unfolded protein response (UPR) is a stress-induced cyto-protective mechanism elicited towards an influx of large amount of proteins in the endoplasmic reticulum (ER). In the present study, we evaluated if AAV manipulates the UPR pathways during its infection. We first examined the role of the three major UPR axes, namely, endoribonuclease inositol-requiring enzyme-1 (*IRE1α*), activating transcription factor 6 (*ATF6*) and PKR-like ER kinase (*PERK*) in AAV infected cells. Total RNA from mock or AAV infected HeLa cells were used to determine the levels of 8 different ER-stress responsive transcripts from these pathways. We observed a significant up-regulation of *IRE1α* (up to 11 fold) and *PERK* (up to 8 fold) genes 12–48 hours after infection with self-complementary (sc)AAV2 but less prominent with single-stranded (ss)AAV2 vectors. Further studies demonstrated that scAAV1 and scAAV6 also induce cellular UPR *in vitro*, with AAV1 vectors activating the *PERK* pathway (3 fold) while AAV6 vectors induced a significant increase on all the three major UPR pathways [6–16 fold]. These data suggest that the type and strength of UPR activation is dependent on the viral capsid. We then examined if transient inhibition of UPR pathways by RNA interference has an effect on AAV transduction. siRNA mediated silencing of PERK and IRE1α had a modest effect on AAV2 and AAV6 mediated gene expression (∼1.5–2 fold) *in vitro*. Furthermore, hepatic gene transfer of scAAV2 vectors *in vivo*, strongly elevated IRE1α and PERK pathways (2 and 3.5 fold, respectively). However, when animals were pre-treated with a pharmacological UPR inhibitor (metformin) during scAAV2 gene transfer, the UPR signalling and its subsequent inflammatory response was attenuated concomitant to a modest 2.8 fold increase in transgene expression. Collectively, these data suggest that AAV vectors activate the cellular UPR pathways and their selective inhibition may be beneficial during AAV mediated gene transfer.

## Introduction

Adeno-associated virus vectors based on serotype (AAV) 2 have shown great promise for therapeutic gene transfer when targeted to immune-privileged sites [1], [2], [3], but their efficacy has been modest when targeted to other tissues such as during hepatic gene transfer [4], [5]. This suggests that cell-specific barriers affect the transduction potential of these vectors. A thorough understanding of the biological interactions between the virus and its host cellular environment is thus necessary to design optimal gene transfer strategies aimed at either in improving their transduction efficiency or in their ability to evade host cellular immune response. Previous studies have demonstrated that several steps in the life cycle of AAV vector influences its transduction efficiency including the receptor-co-receptor binding [6], internalization, intracellular cytoplasmic trafficking to the nuclear membrane [7] and viral uncoating [8]. Following attachment to cell surface receptors [6], AAV2 enters the cell by receptor mediated endocytosis through clathrin and dynamin dependent internalization process [9]. The sub-cellular events after internalization have not been completely elucidated. Recent studies have shown that the virions are trafficked through acidic endocytic compartments followed by retrograde transport to trans-Golgi or endoplasmic reticulum (ER) -Golgi intermediate compartment or the ER [10]. This endosomal trafficking of AAV results in the acidification of its capsid and contributes to its escape into the cytoplasm. It is increasingly clear that each of these intracellular trafficking steps constitute a major rate-limiting step for AAV transduction [11]. For eg., pharmacological inhibition of endosomal acidification by bafilomycin A1 [12], [13] or disruption of golgi apparatus by brefeldin A [13] negatively impacts AAV transduction by 10 to 100 fold, respectively. Remarkably, while the inhibition of ER stress by proteasome inhibitors [14] or in cellular models [15] has been shown to improve the transduction of AAV, conversely, the role of AAV vector load induced stress on ER compartment and its ensuing signalling events are not known.

The ER is the organelle where proteins are modified and folded into their native conformations. While correctly folded proteins are transported further into the *trans*-Golgi network [16], the accumulation of misfolded proteins in the ER, causes stress and leads to activation of a coordinated adaptive program called the unfolded protein response (UPR). The process is initiated by sequential and complex activation of three sensor molecules namely, protein kinase R (PKR)-like ER kinase (PERK), activating transcription factor 6 (ATF6), and inositol-requiring enzyme 1 (IRE1α), whose functions are regulated by immunoglobulin heavy chain binding protein (BiP). The activation of PERK leads to phosphorylation of eukaryotic initiation factor 2α (eIF2 α), leading to a general reduction in protein synthesis as a measure to counteract ER stress [17]. However the mRNA of ATF4, the downstream target of PERK can bypass this translational inhibition since it has upstream open reading frames. This ATF4 translocates to the nucleus activating a set of target genes necessary to bring back the cellular homeostasis [18]. The cellular stress can also translocate ATF6, a transcription factor, to the golgi apparatus, where it is sequentially cleaved by site-1 protease (S1P) and S2P and gets activated. The cleaved ATF6 translocates to nucleus binding to ER-stress response elements (ERSE) and induces transcription of several genes, including *BIP*, *CHOP* (CCAAT/enhancer-binding protein homologous protein) and X-box binding protein 1 (*XBP1*) [19]. When the third arm of UPR, IRE1 is activated by trans-autophosphorylation, its endoribonuclease domain cleaves a 26 nucleotide intron from its target XBP1 (X-box binding protein 1) mRNA, thus performing an unconventional splicing. Spliced XBP1 (sXBP1) protein is a potent transcription factor which then translocates to nucleus to bind to UPR elements (UPREs) and activates many genes that are crucial for restoring cellular homeostasis [20]. This highly regulated UPR response to ER stress reduces the demand on the protein-folding machinery and protects cells from further damage. However, in conditions where the sub-cellular accumulation of the misfolded proteins is beyond the processing capacity by the UPR there is a co-ordinated activation of apoptosis and cell death [21].

A massive influx of exogenous proteins such as in the case of viral infection is also known to trigger UPR, to maintain cellular homeostasis [22]. Several viruses such as herpes simplex virus, cytomegalovirus and others are known to induce ER stress and UPR signalling pathways [23], [24], [25], [26], [27], [28], [29]. While some of them such as influenza virus and rotavirus manipulate these UPR pathways to establish its productive infection [29], [30] many viruses such as Japanese encephalitis virus and Tula virus succumb to its activation due to cross activation of UPREs [22]. NF-κB is a major transcription factor activated in response to UPR signalling that results in immune clearance of the hepatitis B (HbX) and hepatitis C (NS4) viral protein [31], [32], [33]. These examples underscore the critical role played by the UPR signalling in regulating viral infections. In AAV2 mediated gene therapy, the concept of capsid protein dependent immunotoxicity is well documented [5], [34] and several groups have shown that cellular cytoplasmic surveillance mechanisms such as the NF-κB signalling [35], MYD88 pathway or toll-like receptor (TLR-9) [36], [37] signalling influence this process. Since some of these pathways are directly influenced by UPR activation, we hypothesized that AAV2 infection induces ER stress and activates cellular UPR. To test this, our studies were designed to comprehensively analyze the role of the three major UPR signalling arms in the life cycle of AAV vectors both *in vitro* and *in vivo*.

## Materials and Methods

### Ethics Statement

This research involved the use of BALB/c mice. The mice were purchased from Jackson Laboratory (Bar Harbour, ME, USA). All animal experiments were approved and carried out according to the Institutional guidelines for animal care (Christian Medical College, Vellore, India). Studies were conducted on mice housed at 22–24°C in individual ventilated cages. Mice had free access to water and food. All efforts were made to minimize any suffering during our studies.

### Cell Lines and Reagents

The human cervical carcinoma cell line, HeLa, was obtained from the American type culture collection (ATCC, Rockville, MD, USA). The cell line was maintained as monolayer cultures in Iscove’s-modified Dulbecco’s medium (IMDM, Life technologies, Invitrogen, Carlsbad, CA, USA) supplemented with 10% fetal bovine serum and 1% (by volume) of 100× stock solution of antibiotics (10,000 U penicillin+10,000 µg streptomycin). Rabbit monoclonal primary antibodies specific for PERK (C33E10), IRE1α (14C10), Phospho-elF2α (Ser51) (119A11), BiP (C50B12), beta-actin (8H10D10) and the anti-rabbit horse radish peroxidise conjugated IgG secondary antibody were from Cell Signaling Technology®, Inc. (Danvers, MA, USA). ER stress inducing agents tunicamycin and metformin were from Sigma-Aldrich (St Louis, MO, USA) while dithiothreitol (DTT) was procured from Life technologies.

### Generation of Recombinant AAV Vectors

Highly purified stocks of recombinant single stranded (ss) and self complementary (sc) AAV2 vectors or scAAV1 or scAAV6 viruses comprising enhanced green fluorescent protein (EGFP) driven by chicken beta-actin (CB) promoter (a kind gift from Dr Arun Srivastava, University of Florida, Gainesville, USA) were generated by triple transfection of AAV-293 packaging cells (Agilent technologies, Stratagene, La Jolla, CA, USA) using polyethyleneimine (PEI, linear, MW 25,000, Polysciences, Inc, Warrington, USA). Briefly, forty numbers of 150 mm^2^ dishes 80% confluent with AAV 293 cells were transfected with AAV1 or AAV2 or AAV6 rep-cap, transgene (ss or ds AAV2-EGFP) and AAV-helper free (p.helper) plasmids. Cells were collected 72 hours post transfection, lysed and treated with 25 units/ml of benzonase nuclease (Sigma Aldrich). Subsequently, the vectors were purified by iodixanol gradient ultra-centrifugation (Optiprep, Sigma Aldrich) (Zolotukhin *et al.*, 1999) followed by column chromatography (HiTrap Sp or Q column, GE Healthcare, Pittsburgh, PA). The vectors were finally concentrated to a final volume of 0.5 ml in phosphate buffered saline (PBS) using Amicon Ultra 10 K centrifugal filters (Millipore, Bedford, MA). Physical particle titers of recombinant AAV2 stocks were determined by quantitative DNA slot blot analysis [38].

### Recombinant AAV2 Vector Transduction Assays *in vitro*

Approximately 8×10^4^ HeLa cells were seeded in a single well of a 24-well plate and incubated overnight at 37°C. Cells were then mock (PBS)-infected or infected with 5×10^3^ viral genomes (vgs)/cell of ssAAV2 or scAAV2 or scAAV1 or scAAV6 vectors. At various time points after infection [2/6/12/24/48 hours post infection (h.p.i)] the cells were harvested for the different assays described below. The transgene expression was measured in cells that were infected for 48 hours by fluorescence microscopy (Leica DMI6000B, GmbH, Wetzlar, Germany). Images from five visual fields of mock-infected and vector-infected cells were analyzed quantitatively by ImageJ analysis software (NIH, Bethesda, MD, USA). Transgene expression (mean value) was assessed as total area of green fluorescence and expressed as mean pixels per visual field (mean ±SD). HeLa cells pre-treated with optimal concentrations of DTT (2 mM) for 5 h and transduced with AAV vectors served as positive control for UPR activation.

### RNA Interference Mediated Knockdown of PERK and IRE1α Pathways *in vitro*

A pre-validated pool of small-interfering (si) RNAs against PERK and IRE1α [*EIF2AK3_5* (*PERK*; SI02223718), *EIF2AK3_6* (*PERK*; SI02223725), *EIF2AK3_1 (PERK*; SI00069048), *EIF2AK3_10* (*PERK*; SI04438224) and *ERN1_5* (*IRE1*; SI00605248), *ERN1_6* (*IRE1*; SI00605255), *ERN1_17* (*IRE1*; SI04713485), *ERN1_18* (*IRE1*; SI04948839)] was used (Qiagen FlexiTube Gene Solution, Valencia, CA, USA). Cells in the test condition were transfected with 100 nM of test siRNA or scrambled siRNA using Lipofectamine (Life Technologies). Twenty-four hours post siRNA or mock transfection, cells were infected with scAAV2-EGFP. Forty-eight hours post-transduction, the EGFP expression was measured by fluorescence microscopy.

### Hepatic Gene Transfer of Recombinant AAV2 Vectors *in vivo*

All animal experiments were performed according to the Institutional guidelines for animal care specified at Christian Medical College (Vellore, India). BALB/c mice were purchased from Jackson Laboratory (Bar Harbour, ME, USA). For studying the innate immune response against AAV2 vectors, groups of 8–12 weeks-old BALB/c mice were administered with PBS alone (n = 4 animals) or metformin (n = 8 animals, 250 mg/kg body weight) or tunicamycin (n = 4 animals, 1 µg/g body weight) 4 h prior to vector injection. Four animals from each of these groups were then mock-injected (PBS) or injected with ∼1×10^11^ viral genome particles (vg) of scAAV2- EGFP vectors per animal in a 200 µl suspension *via* the tail vein. Twenty-four hours later mice were euthanized and liver samples were collected for further molecular or biochemical analysis.

In our next set of experiments, we wished to evaluate if pharmacological suppression of UPR improves transgene expression from AAV vectors *in vivo*. Three groups (mock, scAAV2 alone and scAAV2+metformin) of animals (n = 4 per group) were used. Animals were either mock (PBS) injected or injected with metformin (250 mg/kg, metformin+AAV2 group) i.p on days −2, −1, and day 0 of gene transfer. At day 0, the animals from the scAAV2 group and scAAV2+metformin groups were injected with ∼1×10^11^ vg of scAAV2- EGFP vectors per animal in a 200 µl suspension *via* the tail vein. The metformin+scAAV2 group of animals received metformin every third day until they were euthanized 4 weeks after gene transfer. Liver lobes from all these animals were collected and analysed for EGFP expression by fluorescence microscopy.

### Real-time Quantitative PCR Analysis of UPR Signalling Pathways

Total cellular RNA was isolated from HeLa cells infected with AAV1 or AAV2 or AAV6 vectors at different time points (2 h, 6 h, 12 h, 24 h and 48 h) using Trizol® reagent (Sigma Aldrich). Similarly, ∼50 mg of liver tissue from the control and test animals was used to isolate hepatic RNA (RNeasy mini kit, Qiagen, Valencia, CA, USA). Approximately 3 µg of the total RNA was reverse transcribed using superscript II first strand cDNA synthesis kit according to the manufacturer’s protocol (Life technologies, Invitrogen). About 1 µl of cDNA was amplified for 6 UPR pathway genes (PERK/BiP/CHOP/ATF6/IRE1/ATF4) using the primers described in table 1 and the data normalized to RPL19 endogenous control gene. A two step PCR reaction was performed in a 10 µl volume, using DyNAmo™ HS SYBR® Green qPCR Kit (Thermo Scientific, Rockford, USA). PCR condition was set at an initial denaturation at 95°C for 15 mins, 40 cycles at 95°C for 10 secs and 60°C for 1 min. Data was captured and analyzed using the Applied Biosystems 7500 Fast Real-Time PCR system SDS 1.4 Software (Life Technologies, Applied Biosystems). The relative gene expression between mock-infected and AAV infected cells was measured by the comparative threshold cycle (ΔΔCt) method and values >2 fold were considered as differentially regulated between the groups.

**Table 1:** Primers used for quantification of UPR signalling pathway genes by real time PCR.

Target gene	Primer sequence
**PERK**	Forward-TCATCCAGCCTTAGCAAACCReverse-ATGCTTTCACGGTCTTGGTC
**BiP**	Forward-CACAGTGGTGCCTACCAAGAReverse-TGTCTTTTGTCAGGGGTCTTT
**CHOP**	Forward-AGCCAAAATCAGAGCTGGAAReverse-TGGATCAGTCTGGAAAAGCA
**ATF6**	Forward-TTGACATTTTTGGTCTTGTGGReverse-GCAGAAGGGGAGACACATTT
**IRE1α**	Forward-CTCTGTCCGTACCGCCCReverse-GAAGCGTCACTGTGCTGGT
**CRT**	Forward-ACAACCCCGAGTATTCTCCCReverse-TGTCAAAGATGGTGCCAGAC
**EDEM**	Forward-GCTCAACCCCATCCACTGReverse-CCAATGCATCAACAAGAGTCA
**ATF4**	Forward-GTCCCTCCAACAACAGCAAGReverse-CTATACCCAACAGGGCATCC
**Rpl19** *	Forward-ATGTATCACAGCCTGTACCTGReverse-TTCTTGGTCTCTTCCTCCTTG

* Primers for Rpl19 gene were from Hiramatsu et al, 2011 [56].

### Reverse Transcription (RT)-PCR to Identify XBP1 Splice Variants

Total RNA isolated at 2 h, 6 h, 12 h, 24 h and 48 h from mock or AAV2 infected HeLa cells was reverse transcribed as described above. Cells treated with DTT for 5 h were used as positive control. Approximately 2 µl of the cDNA was amplified using primers described in table 2 in a ready reaction PCR master mix (ABgene®, Epsom, UK) at a concentration of 1× (68 mM Tris–HCl (pH 8.8), 18 mM (NH_4_)_2_SO_4_, 0.18 mM each of dNTPs, 0.01% (V/V) Tween 20, 2.5 mM Mgcl_2_ and 1.1 units of DNA polymerase). The spliced (267 bp) and unspliced (283 bp) variants of XBP1 were resolved by 2% agarose gel electrophoresis.

**Table 2:** Primers used for detection of XBP1 transcript isoforms by reverse-transcription (RT)-PCR.

Gene*	Primer sequence [56]
XBP1	Forward-TTACGAGAGAAAACTCATGGCC
XBP1	Reverse-GGGTCCAAGTTGTCCAGAATGC

* Primers for XBP1 gene were from Hiramatsu et al, 2011 [56].

### Inflammatory Cytokines and Receptors Pathway Specific RT-PCR Array

For assessing the *in vivo* modulation of inflammatory immune response to AAV2 vectors during UPR inhibition, hepatocyte RNA was isolated from groups of PBS- or metformin injected mice and administered with ∼1×10^11^ vgs of scAAV2- EGFP vectors. The cDNA was profiled by the mouse inflammatory cytokines & receptors profiler array (Qiagen, SABiosciences, Frederick, MD, USA) to determine the relative gene expression of 84 key genes related to innate immune response. The data was acquired using an Applied Biosystems 7500 Fast Real-Time PCR system (Life Technologies, Applied Biosystems). Relative gene expression was measured by the comparative threshold cycle (ΔΔCt) method and analysed by the SABiosciences web based software www.sabiosciences.com/pcr/arrayanalysis.php. Briefly, the fold-change (2^∧^(- Delta Delta Ct)) from the normalized gene expression (2^∧^(- Delta Ct)) in the test sample (metformin+scAAV2 injected group) divided by the normalized gene expression (2^∧^(- Delta Ct)) in the control sample (scAAV2 treated group) was calculated. Fold-change values greater than one indicate a positive- or an up-regulation, while values less than 1 represent down-regulation of test genes. The fold-regulation is equal to the fold-change.

### Immunoblotting

Total protein from HeLa cells transfected with siRNA was isolated as per the manufacturer’s protocol (Cell Signaling Technology Inc, Danvers, MA, USA) in the presence of a protease inhibitor cocktail (Cell signaling). Similarly lysates from HeLa cells infected with ssAAV2 and scAAV2 in triplicates for each of the condition at different time points (2, 6, 12, 24 and 48 h.p.i) were also collected. The protein extracts were boiled for 5 min. under reducing conditions [SDS-sample buffer containing 62.5 mM Tris-HCl (pH 6.8 at 25°C), 2% w/v SDS, 10% glycerol, 50 mM DTT, 0.01% w/v bromo-phenol blue (Cell Signaling)] pooled and stored at −86°C until further analysis. The total protein concentration in the lysate was then determined by the BCA™ protein assay kit (Thermo scientific, Rockford, USA). Equal concentrations of protein lysates (10 µg) were resolved by SDS-PAGE in 4–20% Tris-HCl gradient gels (Biorad Laboratories, Hercules, CA, USA), transferred to Immobilon-P membranes (Millipore, Bedford, MA) and probed with antibodies to PERK, IRE1α, BiP phoshpho-elF2α or β-actin (Cell Signalling) and further by detected by anti-idiotype secondary antibodies. The immuno-reactive bands were visualized using a chemiluminescence detection kit (ECL-Plus, GE healthcare, WI, USA) and documented in ImageQuant 400 imager (GE healthcare). The experiment was then repeated independently. The band intensities from all the test and control conditions was calculated by two independent densitometric scans using ImageJ software (NIH ImageJ, http://rsb.info.nih.gov/nih-image/) and normalized to β-actin protein levels used as loading controls. The average band intensities (+/− SE) were then plotted and generated in Microsoft Excel 2007 version.

## Results

### scAAV2 Vectors Activate the Host Cellular UPR Signaling Pathways *in vitro*

**Figure 1:**
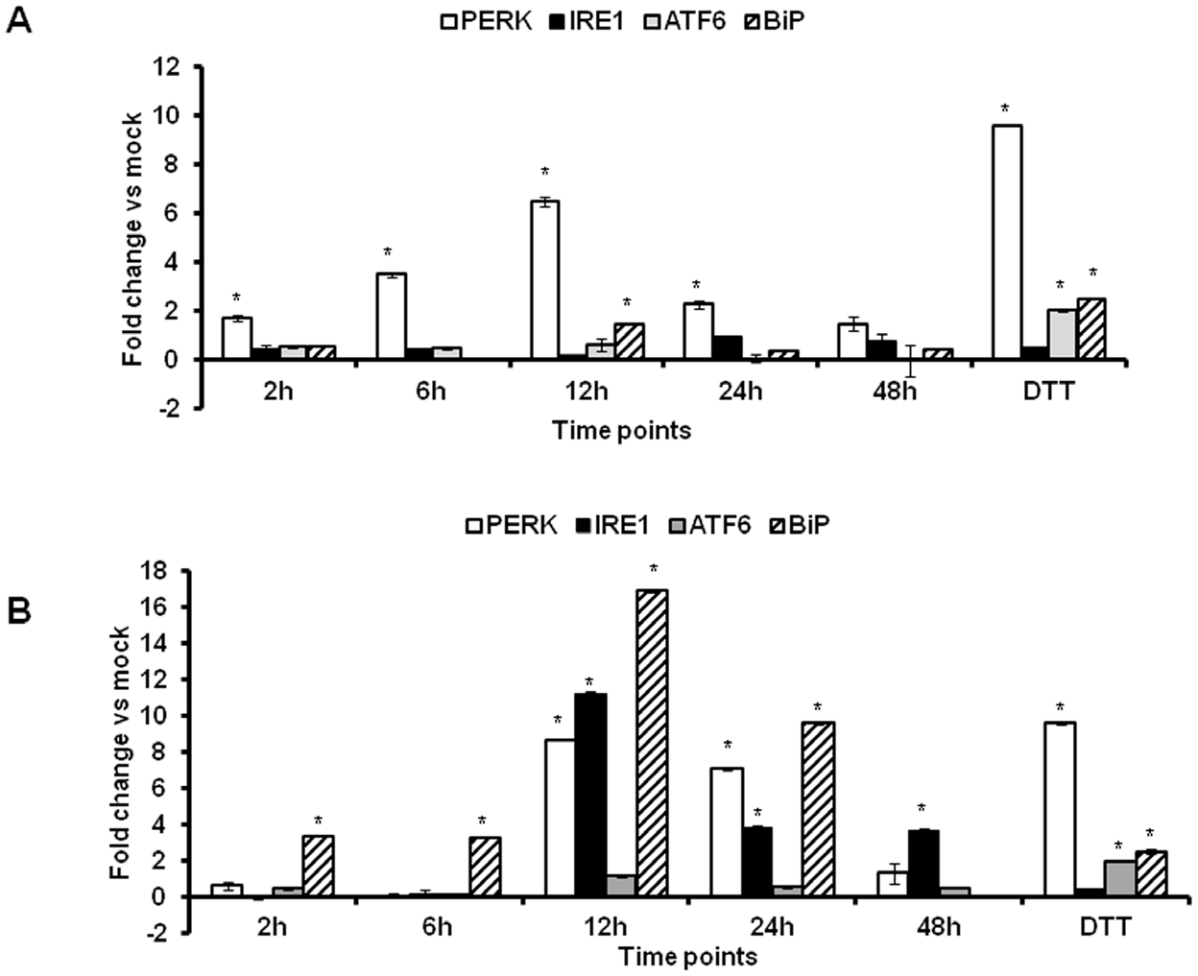
Activation of the cellular unfolded protein response (UPR) pathways against AAV2 vectors *in vitro.* HeLa cells were infected with self-complementary (sc) or single-stranded (ss) AAV2 vectors at an MOI of 5,000 vgs/cell. At various time-points (2/6/12/24/48 h) after infection, total RNA was isolated and the transcript levels of the UPR pathway genes were measured by real-time PCR. Dithiothreitol (DTT, 2 mM) was used as a positive control of UPR activation. **A.** The fold variation in UPR target genes (BiP, PERK, IRE1α and ATF6) expression in cells infected with single-stranded AAV2 at different time points. **B.** The fold variation in UPR target genes (BiP, PERK, IRE1α and ATF6) expression in cells infected with self-complementary AAV2 vectors relative to mock infected cells is shown. *p<0.05 *Vs* mock infected cells.

**Figure 2:**
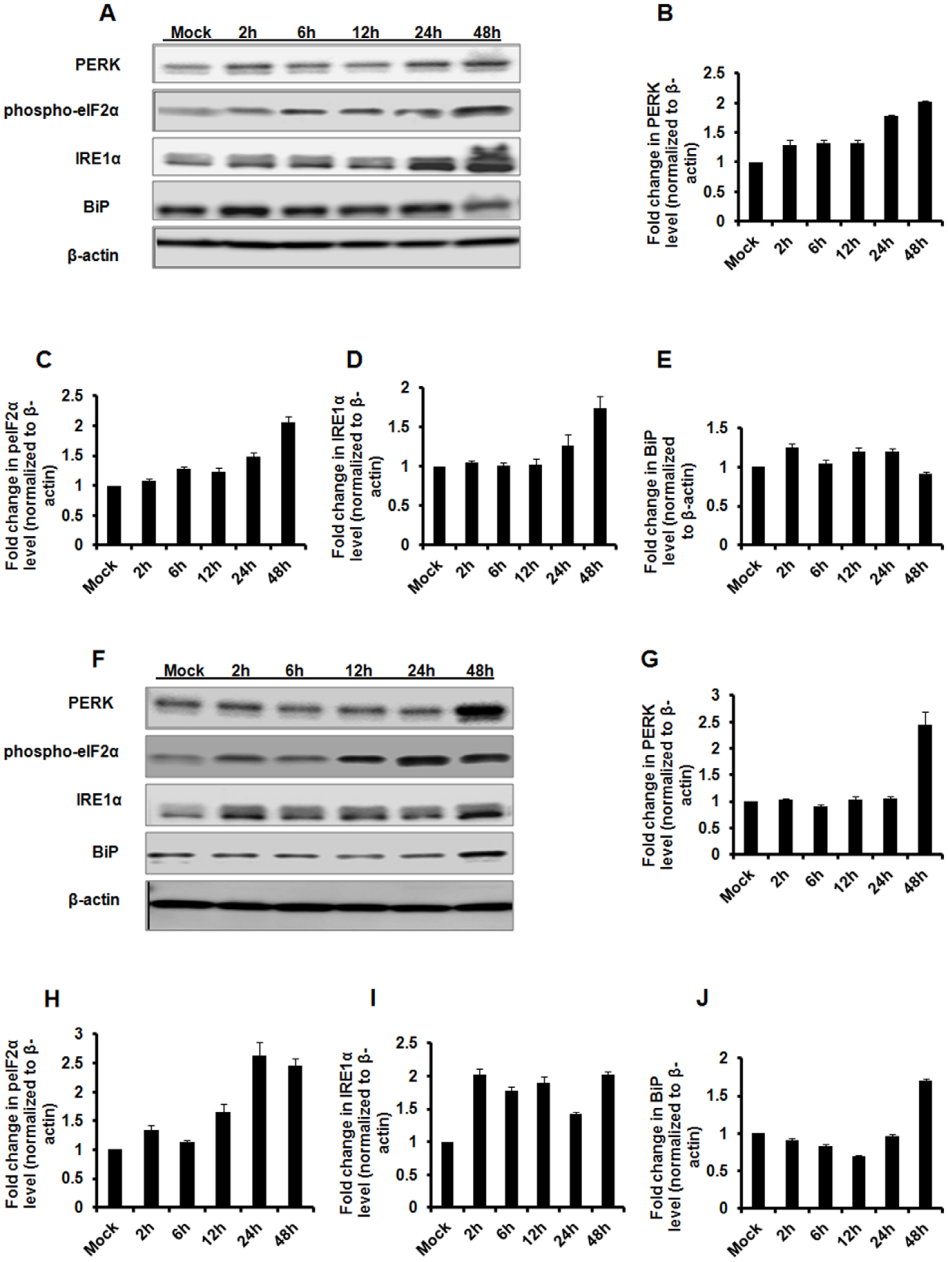
Western blot analysis of UPR activation. HeLa cells were mock-infected or infected in triplicates with 5,000 vgs/cell of AAV2-EGFP vectors. The protein lysates were harvested at 2, 6, 12, 24 and 48 hours post infection for western blot analysis. The protein levels of PERK, phosphorylated-elF2α, IRE1α and BiP at different time points after single stranded AAV2 **(A–E)** or self complementary AAV2 **(F–J)** infection. β-actin was used as a loading control. The band intensities of all the test and control conditions was calculated by two independent densitometric scans using ImageJ software (NIH ImageJ, http://rsb.info.nih.gov/nih-image/) The data is mean +/− S.E from two independent experiments done with protein lysates pooled from triplicate conditions of mock- or AAV infection.

To study if AAV2 elicits an ER stress response, we first examined the major components of UPR signalling pathways during AAV infection. HeLa cells were thus mock- infected or infected with ssAAV2 or scAAV2 vectors at an equal MOI (5×10^3^ vgs/cell) and total RNA was extracted at different time-points, 2, 6, 12, 24 and 48 h.p.i. The UPR target gene expression levels were measured by quantitative PCR at each of the above mentioned time-points. As can be seen in Fig. 1, at 12 h.p.i. compared to mock infected cells, the transcript levels of BiP (16 fold), a major regulator of ER stress and the sensor molecules of the UPR such as PERK (9 fold) or IRE1α (11 fold) were up-regulated against scAAV2 while this activation was less prominent against ssAAV2 vectors. Western blot analysis (Fig. 2) demonstrated a similar increase in UPR pathway proteins in scAAV2 treated cells between 12–48 h.p.i. As can be seen in Fig 2H, components of PERK pathway such as phospho-elF2α levels gradually increased from 2 h.p.i (1.3 fold), 12 h.p.i (1.6 fold), 24 h.p.i (2.6 fold) declining at 48 h.p.i (2.4 fold) while PERK itself was maximally up regulated at 48 h.p.i (Fig 2I). A similar increase (1.4 to 2.0 fold) was seen for IRE1α protein between 2 h.p.i to 48 h.p.i (Fig 2J). However, a less prominent increase in PERK and IRE1α pathways was seen with ssAAV2 vectors (Fig 2C–2D). As would be expected, the levels of UPR pathway proteins seems to gradually increase between 2–48 h.p.i concomitant to peak transcript levels detected at 12 h.p.i with AAV vectors. While the basis for the differential induction of UPR between ssAAV2 and scAAV2 is not clear, it is possible that the UPR signalling pathways in response to scAAV infection may be regulated by toll-like receptor (TLR) family members. It is noteworthy that TLR-9 is known to be activated in response to scAAV2 infection but not against ssAAV2 [36]. The role of TLRs in UPR activation is also well documented [39], [40]. However, this UPR activation is not likely due to the accumulation of the transgene (EGFP) product as HeLa cells transfected with dsAAV-EGFP plasmids did not activate this ER stress response (data not shown).

### Characteristics of UPR Activation against scAAV2 Vectors

**Figure 3:**
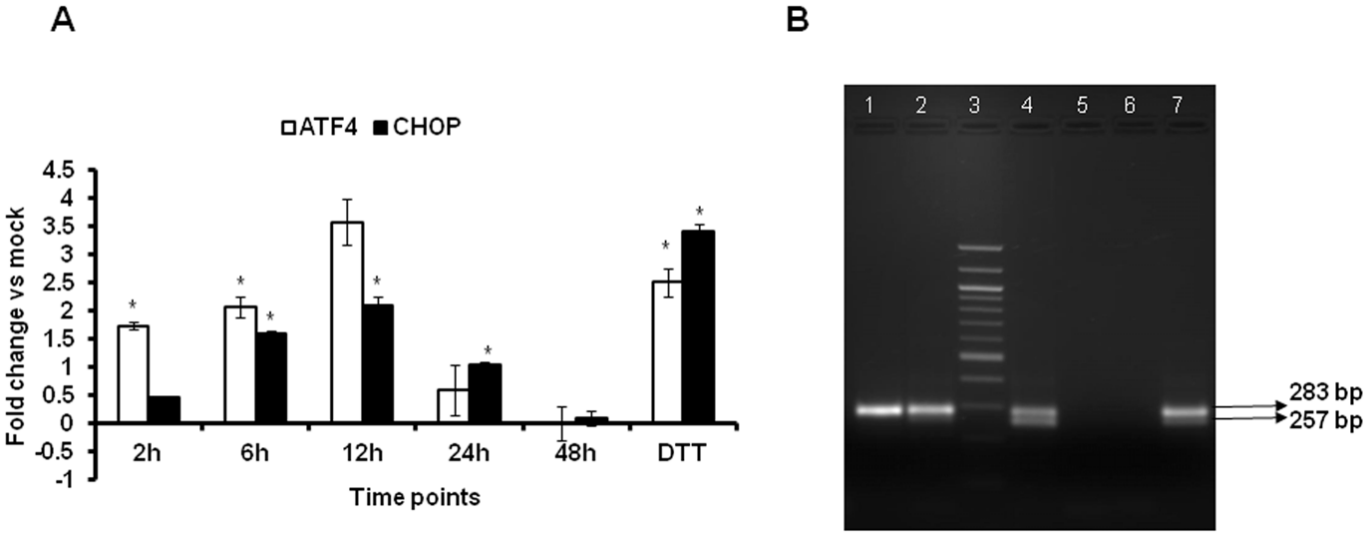
Self-complementary AAV2 infection activates PERK1 and IRE1α pathway and its downstream targets. **A.** Total RNA from HeLa cells mock-infected or infected with of 5,000 vgs/cell of scAAV2-CB-EGFP vectors was used to profile the expression of downstream targets of IRE1α and PERK target genes such as ATF4 or CHOP by real-time PCR analysis at 2, 6, 12, 24 and 48 hours post infection. *p<0.05 *Vs* mock infected cells. **B.** Qualitative reverse-transcription PCR amplification of XBP1 (283 bp) and spliced variant sXBP1 (257 bp) at various time points, 2 h (lane 1), 6 h (lane 2), Molecular weight ladder (lane 3), 12 h (lane 4), 24 h (lane 5) and 48 h (lane 6) analyzed. Dithiothreitol (DTT, lane 7) was used as a positive control of UPR activation.

To confirm the activation of the IRE1α and PERK UPR pathways against scAAV2 vectors, we then profiled their downstream gene targets in HeLa cells *in vitro*. On conditions of ER stress, IRE1α oligomerizes and generates sXBP1. RT-PCR analysis of XBP1 splicing at different time points (2–48 h.p.i.) demonstrated peak levels of sXBP1 transcripts (ratio 0.55-sXBP1/XBP1) at 12 h.p.i (Fig. 3) a time point at which its activator IRE1α transcripts was also maximal (Fig. 1B). A similar induction of the other downstream targets of PERK pathways (ATF4 or CHOP, 3.5 and 2 fold, respectively) was also observed (Fig. 3). Taken together, the maximal increase in IRE1α and PERK gene transcripts as well as the induction of their downstream targets such as sXBP1 or ATF4/CHOP at 12 h mirrors the time-frame at which these vectors move into the ER during their intra-cellular trafficking [11]. A similar wave-like induction of the UPR pathway has been noted for other viruses such as HCV with peak levels of UPR gene transcripts reached between days 3–5, in a manner that coincides with viral replication [41].

### AAV Serotypes 1 and 6 Activate Distinct Arms of UPR Pathways

**Figure 4:**
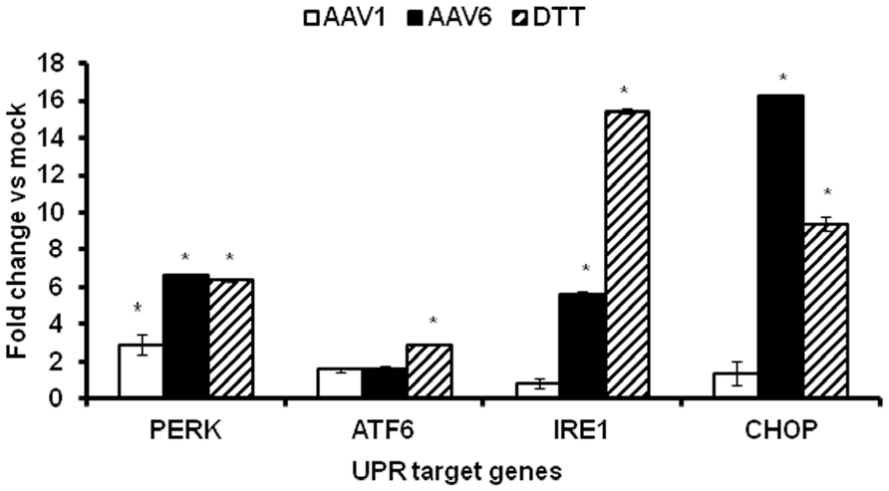
Alternate serotypes AAV 1 and AAV6 induce cellular unfolded protein response. **A.** HeLa cells were infected with 5,000 vgs/cell of scAAV1- EGFP or scAAV6-EGFP vectors under identical conditions. Twelve hours post infection, the differential gene expression of UPR targets were assessed between mock-infected or AAV infected cells. Expression level of PERK, ATF6, IRE1 and CHOP from cells treated with AAV1 and AAV6. DTT (Dithiothreitol) was used as a positive control of UPR activation. *p<0.05 *Vs* mock infected cells.

We then investigated if serotypes other than AAV2 can also modulate UPR signalling pathways *in vitro*. HeLa cells infected with scAAV1 and scAAV6 vectors were studied for UPR activation. Cells infected with scAAV1 vectors had elevated levels of PERK transcripts, ∼3 fold more than mock-infected cells at 12 h.p.i. (Fig. 4). Interestingly, scAAV6 vectors up regulated distinct arms of UPR with significant increase in PERK (7 fold), CHOP (16 fold) and IRE1α (6 fold) genes (Fig. 4). While the scAAV6 mediated increase with PERK transcript levels is comparable to scAAV2 induction of PERK transcripts (7 Vs 8 fold) the strength of IRE1α induction was much lesser for scAAV6 vectors when compared to scAAV2 vectors (6 Vs 11 fold) (Fig. 1B and Fig. 4). It is known that the individual arms of UPR influence the cell’s ultimate fate in response to ER stress [42]. These data show that AAV vectors irrespective of the serotype used perturb ER homeostasis, but lead to distinct UPR signalling signatures that are capsid dependent.

### Molecular Inhibition of PERK and IRE1α Pathways has a Modest Effect on scAAV2 and scAAV6 Mediated Gene Expression *in vitro*

**Figure 5:**
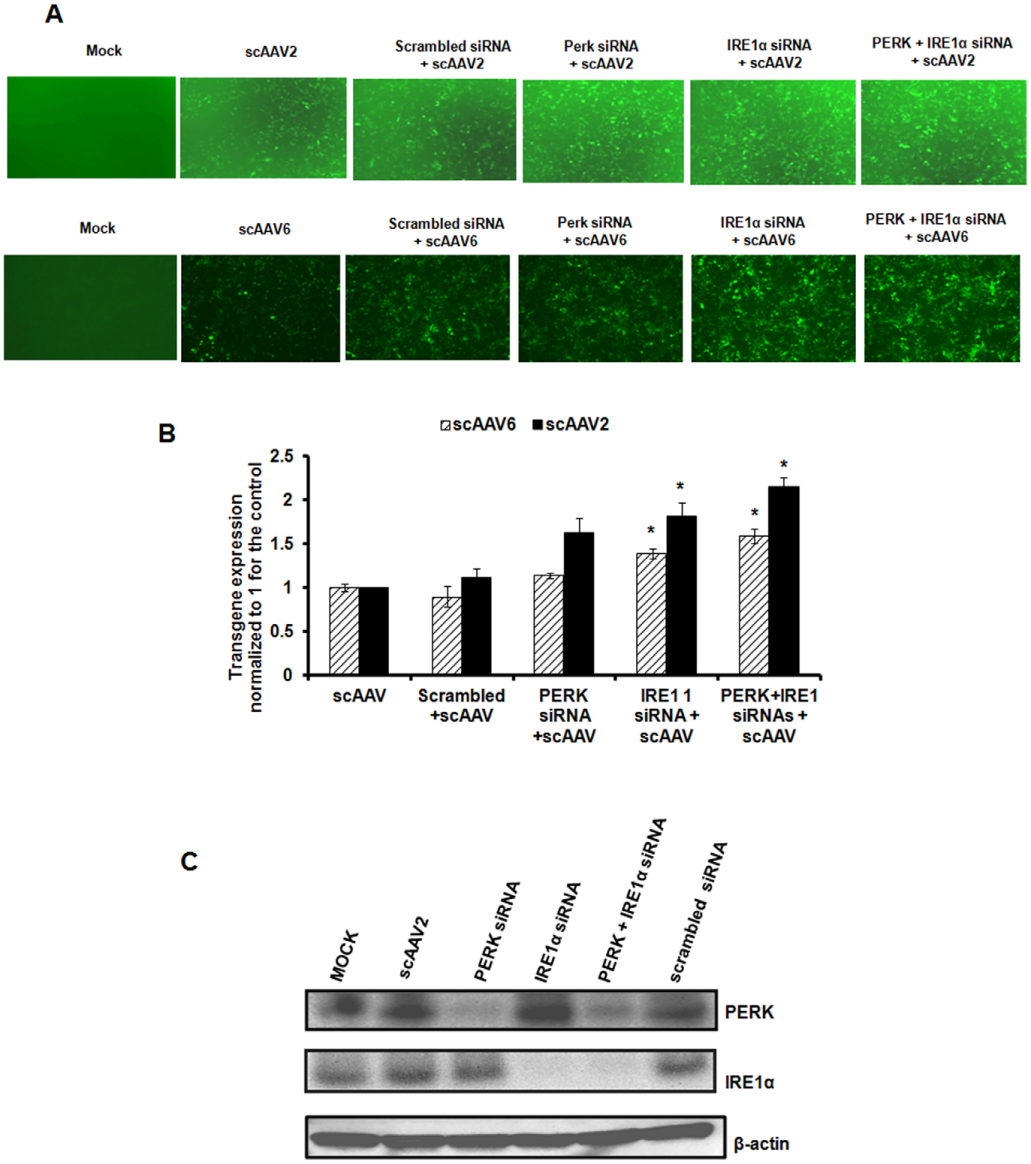
Comparative analysis of AAV mediated transduction efficiency in HeLa cells after siRNA mediated knock down of PERK or IRE1α pathways. **A.** Transgene expression was measured in HeLa cells 48 hrs post-infection with self-complementary AAV2-EGFP or AAV6-EFGP vectors either in the presence or absence of specific siRNA or scrambled siRNA control. **B.** Quantitative analyses of the data from (A) by fluorescence microscopy. Images from five visual fields were analyzed quantitatively by ImageJ analysis software. Transgene expression was assessed as total area of green fluorescence (pixel^2^) per visual field (mean ± SD) and normalized to 1 for the control. Error bars represent standard error and the graph is a representative data set of at least three independent experiments. *p<0.05 *Vs* scrambled siRNA treated cells **C.** Western blot analysis of HeLa cellular extracts following mock (PBS)-infection or infection with AAV vectors, either in the presence or absence of PERK or IRE1α siRNA or scrambled siRNA control. β-actin was used as a loading control.

To test if transient blocking of specific UPR pathways prior to infection with AAV may modulate their transduction efficiency, we knocked down PERK or/and IRE1α by siRNA and measured the EGFP expression from AAV vectors. As can be seen in Fig. 5A,B, the gene expression from scAAV2 upon inhibition of PERK or IRE1α was modestly higher (∼1.6–1.8 fold) compared to the cells transfected with scrambled siRNA. Interestingly, the abrogation of both these pathways together had a similar effect on both AAV2 (2.0 fold) and AAV6 (1.5 fold) transduction, as knockdown of either one of these pathways alone. Western blot analysis of cellular extracts from cells transduced with AAV2 or AAV6 vectors showed marked depletion of PERK and IRE1α protein levels which correlated with a concomitant increase in EGFP expression from these vectors (Fig 5C). The knockdown of PERK pathway alone seems to have only a negligible effect on AAV mediated gene expression. While the basis for this is not clear, it is possible that PERK inhibition cannot completely reverse the protein synthesis block induced by AAV vectors in the transduced cells [51]. These data provide proof-of-concept that UPR repression could modulate the gene expression from AAV vectors.

### scAAV2 Upregulates UPR Genes after Hepatic Gene Transfer *in vivo*

**Figure 6:**
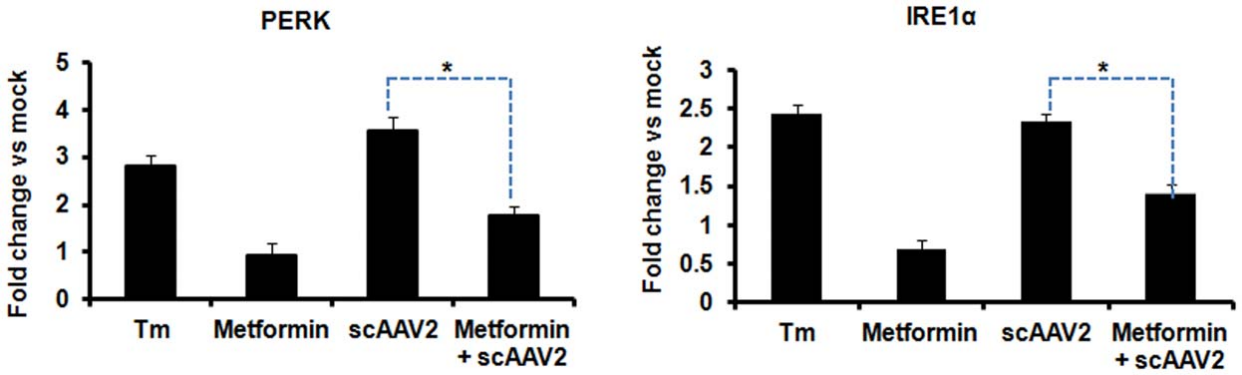
Self-complementary AAV2 mediated hepatic gene transfer in BALB/c mice activates UPR signalling. Groups of mice (n = 4) were injected with 1×10^11^ vg of scAAV2 vgs/animal intravenously with or without prior treatment with the UPR inhibitor, metformin (Met) (250 mg/kg body weight). Animals which received tunicamycin (1 µg/g) were used as positive controls for UPR activation. Twenty four hours after vector injection, the animals were euthanized and hepatic mRNA was assayed for the levels of PERK (**A**) or IRE1α (**B**) genes by real time PCR. *p<0.05 *Vs* AAV2 vector administered mice. Tunicamycin (Tm) injected animals were used a positive control for UPR activation. Mice treated with metformin alone were used as mock control.

To test if AAV2 vectors can also modulate UPR *in vivo*, mice were mock-injected or injected with AAV2 vectors alone or with metformin. Metformin, a UPR inhibitor has been previously shown to block UPR genes in murine models and in cell lines *in vitro* [43], [44]. Compared to the baseline (mock) group (Fig. 6), IRE1α and PERK genes were significantly elevated (2.5–3.5 fold) in mice livers that received scAAV2 vectors at a dose of 1×10^11^ vg/animal. This increased UPR gene expression was comparable to data from mice that received tunicamycin, an ER stress inducer and a UPR activator [45]. However, the elevated UPR transcript levels in AAV2 vector treated mice were significantly attenuated [1.7 fold for PERK and 1.4 fold for IRE1α genes] by pre-administration of the drug, metformin. These results, strongly suggest that hepatic gene transfer of AAV2 vectors induce ER stress and activates UPR genes *in vivo*.

### Pharmacological Inhibition of UPR Attenuates Innate Immune Response and Modestly Improves Transgene Expression from AAV2 Vectors

**Figure 7:**
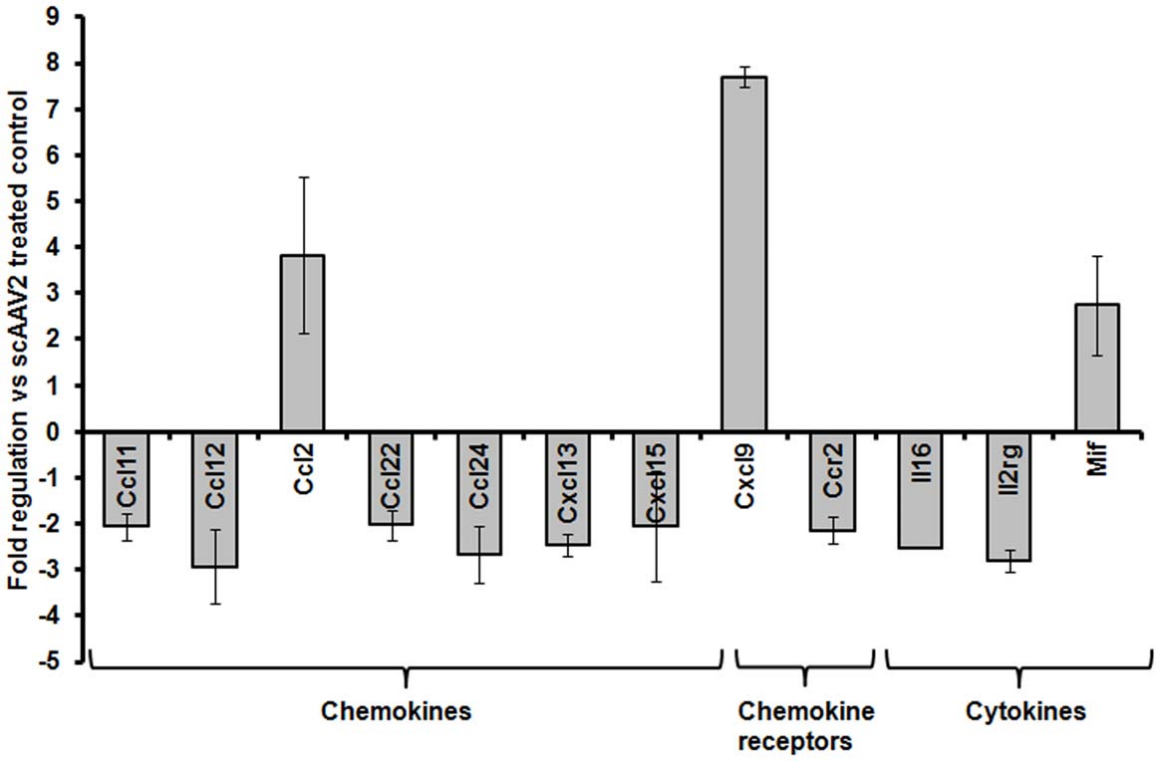
Comparative gene expression profiling of AAV vector-induced inflammatory and immune response markers in the presence or absence UPR inhibitor during hepatic gene transfer *in vivo*. Hepatic gene expression of various inflammatory cytokines in the scAAV2 injected BALB/c mice was measured 24 hours post vector administration. Genes which are significantly different between (2 fold, p<0.05) between mice that received AAV2 and metformin compared to the vector administered group alone, are shown.

Since the role of IRE1α as an activator of NF-κB dependent innate immune response is known [46] and we have previously shown that scAAV2 vectors activate NF-κB pathway [35], we reasoned that blocking UPR signalling may also dampen the innate immune response against AAV vectors. To study this, we compared the expression of various inflammatory/chemokine genes in mice that received scAAV2 vectors alone, compared to mice that had been treated with the UPR inhibitor, metformin, prior to hepatic gene transfer with scAAV2 vectors. As shown in Fig. 7, a variety of genes involved in pro-inflammatory response (interleukin-16 (IL16), IL2 receptor gamma (IL2rg)) as well as other molecules involved in the propagation of chemokine response to AAV (Chemokine (C-C motif) ligand 11 (Ccl11), Chemokine (C-C motif) ligand 12 (Ccl12), Chemokine (C-C motif) ligand 22 (Ccl22), Chemokine (C-C motif) ligand 24 (Ccl24), chemokine C-X-C motif ligand 13 (CXCL13), chemokine C-X-C motif ligand 15 (CXCL15) and chemokine C-C motif receptor 2 (Ccr2) were significantly suppressed by UPR inhibition. Most of these molecules are inflammatory mediators known to attract monocytes and lymphocytes to target tissue. It is well known that key sensors of UPR (PERK, IREα and ATF6) can directly activate NF-κB which in turn can up regulate genes involved in the inflammatory pathway [47], [48].

**Figure 8:**
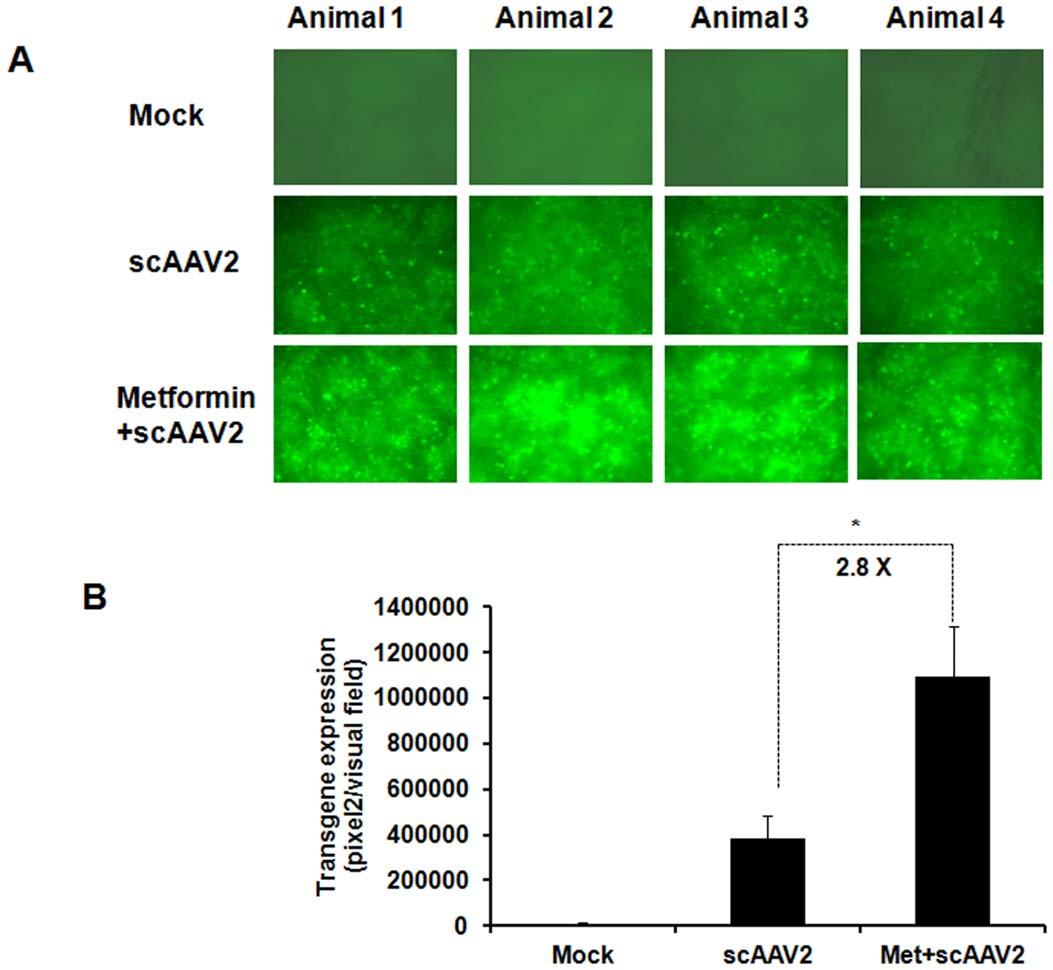
Pharmacological inhibition of UPR increases self-complementary AAV2 mediated transgene expression in vivo. C57/BL6 mice were either mock injected or injected with scAAV2 alone or with metformin and scAAV2 vectors at a dose of 1×10^11^ vgs per mouse. Four weeks later, mice were euthanized and the liver lobes were studied for EGFP expression by fluorescence microscopy. All images were taken at an identical exposure of 576 milliseconds, gain of 1.5 and an intensity of 2. **A.** Representative images from each of the groups. **B.** Images from five visual fields per group were analyzed quantitatively using image-J software. *p<0.05 *Vs* scAAV2 treated mice.

We next studied the effect of pharmacological inhibition of UPR on scAAV2 mediated gene expression. As can be seen in Fig. 8, the EGFP expression in mice treated with metformin and scAAV2 vectors together was modestly higher (∼2.8 fold) than in animals that were administered with AAV vectors alone. These data suggests that transient blocking of UPR may not only suppress the anti-viral innate response but also increase the gene expression during hepatic gene transfer.

## Discussion

Inducible signalling pathways, such as UPR, in response to an active viral infection regulate immediate and long-lived responses necessary for the host cell’s own survival as well as the ability to control the infectious life cycle of the virus. Such responses are probably mediated by changes in viral gene expression and this phenomenon has been reported earlier for hepatitis B and west nile viruses [28], [49]. Since recombinant AAV is replication defective, the vector load is likely to elicit conditions of ER stress and provoke UPR during the course of its infection. Indeed, our studies have demonstrated that AAV vectors activate distinct UPR signalling pathways during their intra-cellular trafficking both *in vitro* and *in vivo*, a molecular pathogenesis hitherto unknown.

One interesting observation from our studies is that the UPR is predominantly activated by scAAV2 than ssAAV2 vectors. During endosomal trafficking, the viral capsid undergoes enormous structural changes, including VP1/VP2 externalization and its disassembly [11]. During this process it has been suggested that the viral particles may be degraded, thereby releasing their DNA contents. And, in case of scAAV vector, due to the mutated inverted terminal repeat sequence at the 3′end of the genome and the space constraints in packaging this genome within the capsid, it has been suggested that these capsids are less stable [50]. This permits substantial release of scAAV genomes during endosomal trafficking and possibly contributes to UPR activation.

The ultimate effect of UPR is a paradox where it can either have a cyto-protective effect by restoring cellular homeostasis or can lead to cell death *via* apoptosis. The activation of the PERK or the combined activation of PERK and IRE1α pathways can lead to enhanced ER protein folding capacity and clearance of misfolded ER proteins or provoke innate immune response against viral proteins. However, depending on the strength of UPR activation, the cells can no longer have the opportunity to restore cellular homeostasis and may eventually lead to apoptosis. It is intriguing though how an identical scAAV genome packaged in either of AAV1 or AAV2 or AAV6 capsid can activate different UPR signalling pathways. One plausible explanation is that these capsids are processed differentially during their intra-cellular trafficking, leading to various degrees of their genome exposure or capsid degradation, which in turn could determine the nature and strength of the UPR. However, further studies are needed to confirm this phenomenon.

Our results suggest that the recombinant AAV vectors in the absence of *in cis* elements such as “rep” and its function may inflict only acute ER stress in infected cells. In our experiments, the induction of various UPR pathways happened between 12–48 h.p.i mimicking acute ER stress conditions. This also explains the modest increase seen in gene expression from scAAV2 or scAAV6 vectors, despite ∼80–100% constitutive knockdown of PERK and IRE1α pathways *in vitro*. These data are in agreement with previous observation in a cystic fibrosis transmembrane conductance regulator misfolded variant cellular model of constitutive ER stress, where activating or blocking the IRE1α pathway did not have any major effect on AAV transduction [52].

Our studies also demonstrate that the acute ER stress induced by scAAV2 facilitates cross-talk between UPR and innate immunity, as has been recently described for several viruses [27], [53], [54]. In particular the link between IRE1α/sXBP1 driven activation of janus kinase (JNK) or phosphotidyl inositol 3-kinase (PI3K) pathway or mitogen associated protein kinase pathway (MAPK) that mediate a NF-κB dependent activation of anti-viral innate immune response is being increasingly recognized [47], [55]. Our previous studies have documented that recombinant AAV vectors activate the classical NF-κB pathway during their cytosolic entry in the acute phase of AAV infection (<2 hrs), and leads to cross activation of alternative NF-κB pathway (>8 hrs) manifesting as a trigger for several of the pro-inflammatory cytokines (IL1α, IL6), TNFα, IL12α, keratinocyte-derived chemokine (KC) and regulated upon activation, normal T-cell expressed and secreted (RANTES) [35]. In line with these observations, we have identified in this study that several of the NF-κB dependent pro-inflammatory genes were upregulated during the course of UPR induction to scAAV2 vectors. Furthermore, the selective ablation of UPR *in vivo,* attenuated both the hepatic UPR and the pro-inflammatory state and improved the transgene expression, suggesting that transient suppression of UPR pathways prior to AAV vector administration might be beneficial.

In conclusion, our study demonstrates that recombinant AAV vectors activate distinct pathways of the cellular UPR and highlights the UPR system as a possible target to attenuate the host inflammatory response against these vectors. Further studies are warranted to dissect the intra-cellular signalling events post-UPR induction, in order to better understand the molecular pathogenesis of AAV vectors during their endosomal processing.
